# Methylsulfonylmethane Inhibits RANKL-Induced Osteoclastogenesis in BMMs by Suppressing NF-κB and STAT3 Activities

**DOI:** 10.1371/journal.pone.0159891

**Published:** 2016-07-22

**Authors:** Youn Hee Joung, Pramod Darvin, Dong Young Kang, Nipin SP, Hyo Joo Byun, Chi-Ho Lee, Hak Kyo Lee, Young Mok Yang

**Affiliations:** 1 Department of Pathology, School of Medicine, Institute of Biomedical Science and Technology, Konkuk University, Seoul, 143-701, Republic of Korea; 2 Department of Food Science and Biotechnology of Animal Resources, Konkuk University, Seoul, 143-701, Republic of Korea; 3 Department of Animal Biotechnology, Chonbuk National University, Jeonju, 561-756, Republic of Korea; Faculté de médecine de Nantes, FRANCE

## Abstract

Osteoclast differentiation is dependent on the activities of receptor activator NF-kB ligand (RANKL) and macrophage colony-stimulating factor (M-CSF). Given that RANKL plays a critical role in osteoclast formation and bone resorption, any new compounds found to alter its activity would be predicted to have therapeutic potential for disorders associated with bone loss. Methylsulfonylmethane (MSM) is a naturally occurring sulfur compound with well-documented anti-oxidant and anti-inflammatory properties; currently its effects on osteoclast differentiation are unknown. We sought to investigate whether MSM could regulate osteoclastogenesis, and if so, its mechanism of action. In this study, we investigated the effects of MSM on RANKL-induced osteoclast differentiation, together with STAT3’s involvement in the expression of osteoclastic gene markers. These experiments were conducted using bone marrow derived macrophages (BMMs) and cell line material, together with analyses that interrogated both protein and mRNA levels, as well as signaling pathway activity. Although MSM was not toxic to osteoclast precursors, MSM markedly inhibited RANKL-induced TRAP activity, multinucleated osteoclast formation, and bone resorptive activity. Additionally, the expression of several osteoclastogenesis-related marker genes, including TRAF6, c-Fos, NFATc1, cathepsin K, and OSCAR were suppressed by MSM. MSM mediated suppression of RANKL-induced osteoclastogenesis involved inhibition of ITAM signaling effectors such as PLCγ and Syk, with a blockade of NF-kB rather than MAPK activity. Furthermore, MSM inhibited RANKL-induced phosphorylation of STAT3 Ser^727^. Knockdown of STAT3 using shRNAs resulted in reduced RANKL-mediated phosphorylation of Ser^727^ STAT3, and TRAF6 in cells for which depletion of STAT3 was confirmed. Additionally, the expression of RANKL-induced osteoclastogenic marker genes were significantly decreased by MSM and STAT3 knockdown. Taken together, these results indicate that STAT3 plays a pivotal role in RANKL-induced osteoclast formation, and that MSM can attenuate RANKL-induced osteoclastogenesis by blocking both NF-kB and STAT3 activity.

## Introduction

Bone remodeling describes the restructuring of existing bone, which is a delicately controlled balance between bone formation by osteoblasts and resorption by osteoclasts [1]. An imbalance in these processes can lead to excessive osteoclast-induced bone resorption, which causes rheumatoid arthritis and osteoporosis, and can encourage cancer metastases to the bone [2]. Osteoclasts are specialized bone-resorbing cells regulated by osteoblast through the synthesis of macrophage colony-stimulating factor (M-CSF) and receptor activator of NF-κB ligand (RANKL) [2,3]. RANKL-induced activation of RANK causes TNF receptor-associated factor 6 (TRAF6) recruitment in osteoclast precursor cells [4] and the sequential activation of mitogen-activated protein kinases (MAPKs) involving extracellular signaling-related kinase (ERK), p38, and Jun N-terminal kinase (JNK), and transcription factors such as nuclear factor-kappa B (NF-κB), activating protein 1 (AP-1), nuclear factor of activated T cells (NFATc1), and c-Fos [5]. The activation of these signaling effectors induces the expression of osteoclastic genes such as tartrate-resistant acid phosphatase (TRAP), cathepsin K (Cts K), and matrix metalloproteinase 9 (MMP-9), whose activities result in the development of multinucleated bone-resorbing osteoclasts [5,6].

The family of signal transducer and activator of transcription proteins (STATs) play a pivotal role in growth factor, prolactin, and various cytokine signaling pathways [7]. Recent evidence suggests that STATs, particularly STAT5b, play a central role in growth hormone (GH) signaling and osteoblast differentiation [8]. This finding is supported by our recent *in-vitro* studies showing that methylsulfonylmethane (MSM) enhanced GH-induced osteoblast differentiation via persistent activation of the Jak2-STAT5b signaling pathways [8]. Many studies have demonstrated the importance of STAT3 in bone physiology, with RANKL-mediated osteoclastogenesis diminished by the protein inhibitor of activated STAT3 (PIAS3) [9]. Indeed, recent data demonstrated a dual role for STAT3 depending on cell type (osteoblast or osteoclast) and its phosphorylation status [10].

Sulfur is an essential mineral needed for the biosynthesis of sulfur-containing amino acids, oxygen transport, and in the biosynthesis of various structural and functional proteins including collagen. MSM is an organic sulfur compound found in various fruits, vegetables, grains, and animals including humans [11]. MSM is bioavailable form of dietary sulfur; hence it can resolve the sulfur deficiencies and improve cartilage formation. Nevertheless, the effect of MSM on RANKL-induced osteoclastogenesis has yet to be determined.

In this study, we intended to clarify the anti-osteoclastogenic effect of MSM on RANKL-induced osteoclastogenesis in bone marrow macrophages (BMMs). In addition, we investigated whether STAT3 is directly involved in RANKL-induced osteoclastic marker gene expression. Our study provides important insights into the involvement of MSM-dependent STAT3 in RANKL-induced osteoclastogenesis.

## Materials and Methods

### Ethics Statement

This study was approved by the Institutional Animal Care and Use Committee, Konkuk University (Seoul, Korea); all procedures and routine animal care were in accordance with institution guidelines.

### Reagents and Antibodies

Modified eagle’s medium (α-MEM), Dulbecco’s modified eagle’s medium (DMEM), trypsin-EDTA, and fetal bovine serum (FBS) were obtained from Gibco-BRL (Grand Island, NY). Recombinant mouse M-CSF and RANKL were obtained from Peprotech (Rocky Hill, NJ, USA). Rabbit polyclonal antibodies specific for phospho-ERK, phospho-JNK, phospho-p38, phospho-PLCγ2, phospho-Syk, IκB, phospho-IKK (Ser176/180), phospho-STAT3 (Ser727), and phospho-Gab2 were purchased from Cell Signaling Technology (Danvers, MS, USA). Mouse monoclonal antibodies specific for NFATc1, TRAF6, cathepsin K, c-Fos, STAT3, TBP, and NF-κB were bought from Santa Cruz Biotechnology (Santa Cruz, CA, USA). Anti-actin antibody, β-glycerophosphate disodium salt hydrate, MSM, ascorbic acid phosphate, 3-(4,5-dimethylthiazol-2-yl)-2,5-diphenyl tetrazolium bromide (MTT), and L-glutamine were purchased from Sigma Chemical Co. (St. Louis, MO). A bone resorption assay kit was purchased from COSMO BIO Co. (Tokyo, Japan). The electrophoretic mobility shift assay (EMSA) kit and oligonucleotide probes (NF-κB) were purchased from Panomics (Redwood City, CA). The RNeasy mini kit was purchased from Qiagen (Hilden, Germany) and enhanced chemiluminescence (ECL) plus detection kit from Amersham Pharmacia Biotech. (Piscataway, NJ).

### Osteoblast Differentiation Protocol

Primary mesenchymal stem cells were prepared from femur and tibia of 6-week-old BALB/c mice (Orient Bio, Gyeonggi-Do, Korea). Bone marrow was flushed out, then cultured, in α-MEM containing 10% FBS. Osteoblastic differentiation was performed as described [8].

### Preparation of Bone Marrow-Derived Macrophages, Osteoclast Differentiation, TRAP Activity Assays and Staining

All mice were sacrificed under the CO_2_ chamber, tibiae and femurs of 6-week-old BALB/c mice were used to obtain bone marrow cells. Residual cells, following the removal of red blood cells, were plated in 100-mm culture dishes and cultured in α-MEM containing 10% FBS, 100 U/ml penicillin, and 100 μg/ml streptomycin. After incubation at 37°C for 1 day, the media for non-adherent cells was supplemented with 30 ng/ml M-CSF. 3 days later, adherent cells (now bone marrow macrophages (BMMs)) were harvested. BMM were seeded into a 96-well plate and cultured with 100 ng/ml RANKL and 30 ng/ml M-CSF for three to five days, with or without MSM. After OC differentiation, the cells were washed and then fixed with 4% paraformaldehyde, and stained for TRAP using leukocyte acid phosphatase cytochemistry kit (Sigma-Aldrich, MO, USA). Mature osteoclasts were categorized as TRAP-positive multinucleated cells with three or more nuclei. Osteoclast counts were conducted using a microscope. To measure TRAP activity, permeabilized cells were treated with 100 μl of citrate buffer containing 10 mM sodium tartrate and 5 mM *p*-nitrophenyl phosphate. After incubation at room temperature, cells mixes were transferred to fresh plates containing 50 μl of 0.1 N NaOH and absorbance read at 405 nm.

### Cell Viability Assay

BMMs (1×10^4^ cells/well) were cultured in 96-well plates for 24 h following incubation with 30 ng/ml M-CSF. Cells were exposed to the indicated concentrations of MSM (25–100 mM) in the presence of 30 ng/ml of M-CSF and 100 ng/ml of RANKL. Cell viability was determined over 4 days using the MTT (5 mg/ml) and Alamar blue assay. All experiments were performed in triplicates and measured three times.

### Bone Resorption Assay

BMMs (1×10^4^ cells/well) were plated on the fluoresceinamine-labeled chondroitin sulfate (FACS)-labeled CaP-coated plates (COSMO BIO Co., Japan). Cells were cultured for 5 days in α-MEM supplemented with 30 ng/ml of M-CSF and 100 ng/ml of RANKL, with or without MSM. On day 5, 100 μl of the conditioned medium was transferred from each well into the wells of a 96-well plate (with a black plate used for fluorescence measurements). Bone resorption assay buffer was added to each well and mixed. An excitation wavelength of 485 nm, with emission at 535 nm was used for fluorescence measurements.

### Western Blot Analyses

BMMs were stimulated by the addition of RANKL (100 ng/ml) for 10 min with or without MSM pretreatment for 1 h. Cells were lysed in RIPA lysis buffer (50 mM Tris-HCl, pH 7.5, 5 mM EDTA, 150 mM NaCl, and 1% Triton X-100) containing 1X BD Baculogold protease inhibitor cocktail (BD Bioscience, CA) and 1X PhosSTOP phosphatase inhibitors. Cytosolic and nuclear proteins were prepared using nuclear extraction reagents (Panomics, Freemont, CA) according to the manufacturer’s instructions. Protein concentrations were then determined using the Coomassie Protein Assay (Pierce, Rockford, IL) and equal amounts of proteins were then separated in a 10% SDS-PAGE, and electro-blotted to nitrocellulose membranes. Membranes were blocked with 5% non-fat milk in T-TBS buffer (20 mM Tris-HCl pH 7.6, 137 mM NaCl, 0.1× Tween 20) and incubated overnight at 4°C with primary antibodies (anti-TRAF6, c-Fos, NFATc1, CatK, p-ERK, p-JNK, p-38, p-Gab2, p-PLCγ2, p-Syk, p-IKK (Ser176/180), IκB, NF-κB, TBP, or β-actin). The membranes were then washed in T-TBS and incubated with the appropriate secondary antibody HRP-conjugate (1:1000) and developed using the ECL PLUS kit.

### EMSA

BMMs were stimulated by the addition of RANKL (100 ng/ml) for 10 min with or without MSM pretreatment for 1 h. Nuclear extracts were prepared using nuclear extract kit. NF-κB DNA binding activity was detected by EMSA, in which a DNA probes, used to bind active NF-κB protein in nuclear extracts. The treated and untreated nuclear extracts were incubated with a biotin-labeled transcription factor (TF-NF-κB) probe and then resolved on a non-denaturing 6% PAGE gel. Following this, the proteins were transferred to a nylon membrane and detected using chemiluminescence.

### Transfection of STAT3 Short Hairpin RNA (shRNA)

RAW 264.7 cells were transfected with 1 μg of STAT3 or non-target shRNA plasmid (Santa Cruz Biotechnology) using transfection reagent (Santa Cruz Biotechnology), in accordance with manufacturer’s instructions. Two days later, cells were stimulated with 100 ng/ml of RANKL for 15 min, with or without MSM pretreatment for 1 h. The cells were harvested for western blot and real-time PCR.

### RT-PCR

Bone marrow mesenchymal stem cells were cultured in α-MEM containing 10% FBS. On day 6, the medium was supplemented with 10 mM sodium β-glycerophosphate and 50 μg/ml ascorbic acid to initiate osteoblast differentiation. Medium was replaced every two to three days. mRNA expression analyzed 21 days after treatment with 20 mM MSM. BMMs were seeded in 6 cm dishes (2x10^6^ cells/dish) and cultured in α-MEM with 10% FBS and 30 ng/ml M-CSF. Then, the cells were stimulated by the addition of RANKL (100 ng/ml) for 10 min with or without pretreatment of MSM for 1 h at 37°C and 5% CO_2_. Using RNeasy Mini kit (Qiagen) the total RNA was prepared. Equal amount of RNA were reverse transcribed using the AccuPower RT PreMix kit (Bioneer) according to the manufacturer’s instructions. The PCR primer sequences were as follows: RANKL, sense: 5′-GCGTCTGTTCCTGTACTTTCGAGCG -3′, antisense: 5′-TCGTGCTCCCTCCTTTCATCAGGTT-3′; M-CSF, sense: 5′-GAGAAGA CTGATGGTACATCC-3′, antisense: 5′-CTATACTGGCAGTTCCACC-3′; OPG, sense: 5′-TGG AGATCGAATTCTGCTTG-3′, antisense: 5′-TCAAGTGCTTGAGGGCATAC-3′; TRAP, sense: 5′-ACTTCCCCAGCCCTTACTAC-3′, antisense: 5′-TCAGCACATAGCCCACA CCG-3′; c-Fos, sense: 5′-CTGGTGCAGCCCACTCTGGTC-3′, antisense: 5′-CTTTCAGCAGA TTGGCAA TCTC-3′; NFATc1, sense: 5′- CAACGCCCTGACCACCGATAG-3′, antisense: 5′-GGCTGC CTTCCGTCTCATAGT-3′; OSCAR, sense: 5′-CTGCTGGTAACGGATCAGCTCC CCAGA-3′, antisense: 5′-CCAAGGAGCCAGAACCTTCGAAACT-3′. The PCR reaction was as follows; 30 cycles at 94°C for 45 seconds, 60°C for 45 seconds, and then 72°C for 1 min. Following amplification, PCR products were analyzed using 1.2% agarose gel containing ethidium bromide and visualized under ultraviolet illumination.

### Gene Expression by Real-Time PCR Analyses

RAW264.7 cells were transfected with STAT3 shRNA or non-targeting shRNA for 48 h and then stimulated with RANKL (100 ng/ml) for 24 h. Total RNA was extracted from transfected RAW264.7 cells after exposure to 50 mM MSM; One microgram of total RNA was employed for cDNA synthesis using the AccuPower RT PreMix kit according to the manufacturer’s instructions. qPCR were carried out in 20 μl solutions, containing 1X FastStart DNA Master SYBR (Roche), 25 mM MgCl_2_, diluted forward and reverse primers, and cDNA. The primer sequences were as follows: STAT3, sense: 5′-AATGGAAATTGCCCGGATC-3′, antisense: 5′-AGGCG AGACTCTTCCCACAG-3′; NFATc1, sense: 5′-CCGTTGCTTCCAGAAAATAACA-3′, antisense: 5′-TGTGGGATGTGAACTCGGAA-3′; TRAP, sense: 5′-CCATGCCAAAGAGATC GCC-3′, antisense: 5′-TCTGTGCAGAGACGTTGCCAAG-3′; OSCAR, sense: 5′-CTGCTG GTAACGGATCAGCTCCCCAGA-3′, antisense: 5′-CCAAGGAGCCAGAACCTTCGAAAC T-3′; c-Fos, sense: 5′-CGCAGAGCATCGGCAGAAGG-3′, antisense: 5′-TCTTGCAGGCAG GTCGGTGG-3′; MMP-9, sense: 5′-CCTGCCAGTTTCCATTCATC-3′, antisense: 5′-GCCA TTCACGTCGTCCTTAT-3′; GAPDH, sense: 5-GGGCATCT TGGGCTA CAC-3, antisense: 5-GGTCCAGGGTTT CTTACTCC-3. The cycling conditions were 40 cycles of two-step cycling program involving a denaturation step at 95°C for 10 sec and a combined annealing/extension step at 60°C for 20 sec. The threshold cycle (Ct) value was calculated from amplification plots. Data were analyzed using the ΔΔCt relative quantification approach. Control calibration was with a pool of reverse transcribed samples, each normalized to an internal control of GAPDH. Each sample was run in triplicate, with data expressed relative to calibrated controls at each time point.

### Statistical Analyses

All data values were expressed as mean ± SEM. Statistical analysis was done with the student’s t-test or analysis of variance (ANOVA) followed by Duncan’s multiple range test using the SAS 9.3 software. A value of P < 0.05 was considered as significant.

## Results

### MSM Suppresses RANKL-Induced Osteoclastogenesis in BMMs

**Fig 1:**
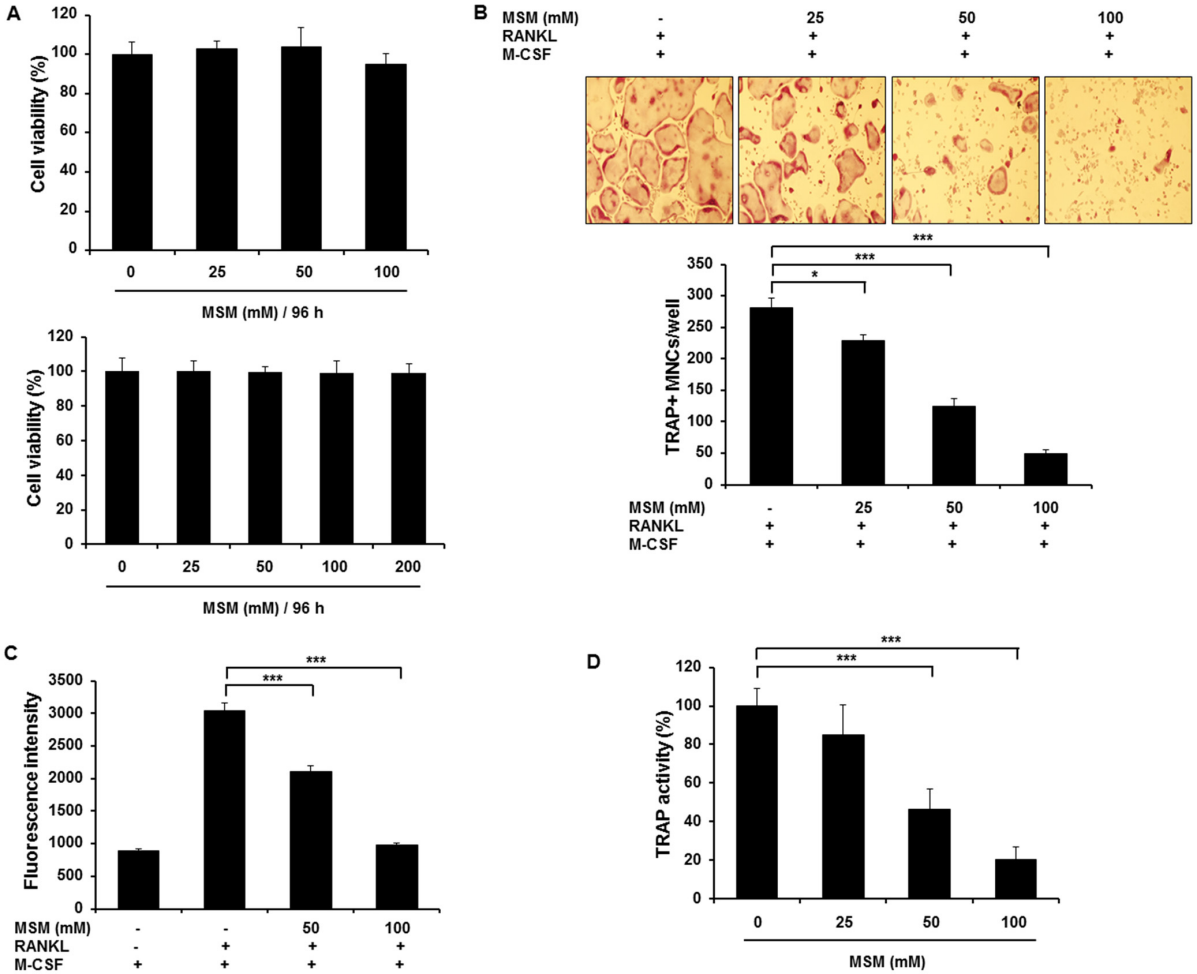
MSM suppresses RANKL-induced osteoclastogenesis in BMMs. BMMs were cultured in the presence of M-CSF (30 ng/ml) and RANKL (100 ng/ml) for 4 days with or without MSM as indicated. (A) Cell viability was evaluated by MTT and Alamar blue assay. (B) TRAP staining with light microscopy enabled TRAP-positive multinucleated cells (MNCs were categorized as containing ≥ 3 nuclei) to be quantified. (C) Quantitative assessment of TRAP activity. (D) Bone resorption activity was evaluated by fluorescence intensity. Data shown are representative of three independent experiments. Asterisks indicate a significant increase by ANOVA (*p <0.05, ***p < 0.001).

BMMs were exposed to various concentrations of MSM for 96 h, then cytotoxicity assessed by MTT assay. As shown in Fig 1A, MSM did not affect the viability of osteoclast precursors. To investigate the direct role of MSM in osteoclast differentiation, we examined its effect on RANKL-induced osteoclast formation using mouse BMMs. BMMs exposed to RANKL and M-CSF efficiently differentiated into multinuclear osteoclasts, while MSM exposure dose-dependently diminished the formation of TRAP-positive multinucleated cells, inhibiting osteoclast formation (Fig 1B). Similarly, MSM decreased RANKL-induced TRAP activity of osteoclasts in a dose-dependent manner (Fig 1D). We then investigated the effect of MSM on RANKL-induced bone resorption during osteoclastogenesis in BMMs. 100 mM MSM suppressed RANKL-induced bone resorption by 75% (Fig 1C). These data indicate that MSM exerts an inhibitory effect on RANKL-induced osteoclastogenesis.

### MSM Suppresses RANKL-Induced Osteoclast Marker Gene and Protein Expression

**Fig 2:**
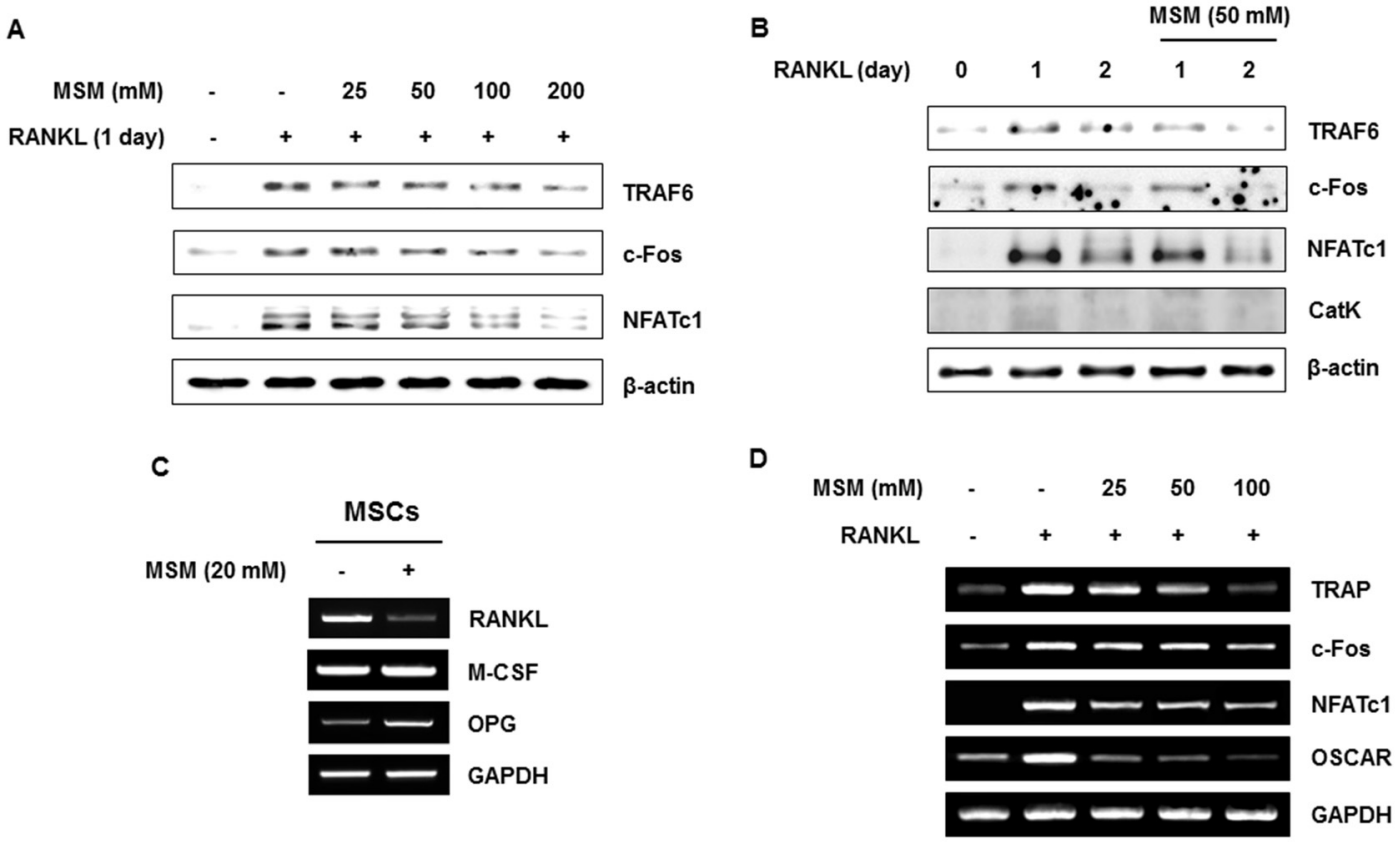
MSM suppresses RANKL-induced osteoclast marker gene and protein expression. BMMs were cultured in the presence of M-CSF (30 ng/ml) and RANKL (100 ng/ml) for the indicated number of days, with or without various concentrations of MSM. (A and B) Expression levels of the RANKL-induced osteoclast marker proteins examined by western blot analyses. (C) Expression of mRNAs for RANKL and OPG in bone marrow mesenchymal stem cells (MSCs). (D) Expression of mRNA for RANKL-induced osteoclast marker genes examined by RT-PCR analyses. Beta-actin and GAPDH were used as loading controls. Data shown are representative of three independent experiments.

To further elucidate the role of MSM in osteoclast differentiation, we examined its effect on the expression of osteoclast marker genes during RANKL-induced osteoclastogenesis by western blot and RT-PCR analyses. Our data revealed that MSM suppressed RANKL-induced TRAF6, c-Fos, NFATc1, and Cts K protein concentration in a dose-dependent manner, and at most time points (Fig 2A and 2B). Furthermore, MSM inhibited mRNA levels for TRAP, c-Fos, NFATc1, and OSCAR (Fig 2C), suggesting that MSM inhibits osteoclast formation, which is regulated by the RANKL and Ca^2+^ signaling pathway. An increased RANKL/osteoprotegerin (OPG) ratio promotes the differentiation of osteoclasts and drives bone resorption. As shown in Fig 2D, the RANKL/OPG ratio in the MSC culture medium supplemented with MSM was reduced in comparison to controls. MSM inhibited the expression of RANKL mRNA in osteoblasts, while promoting the expression of OPG.

### MSM Inhibits the RANKL-Induced Signaling Pathway in BMMs

**Fig 3:**
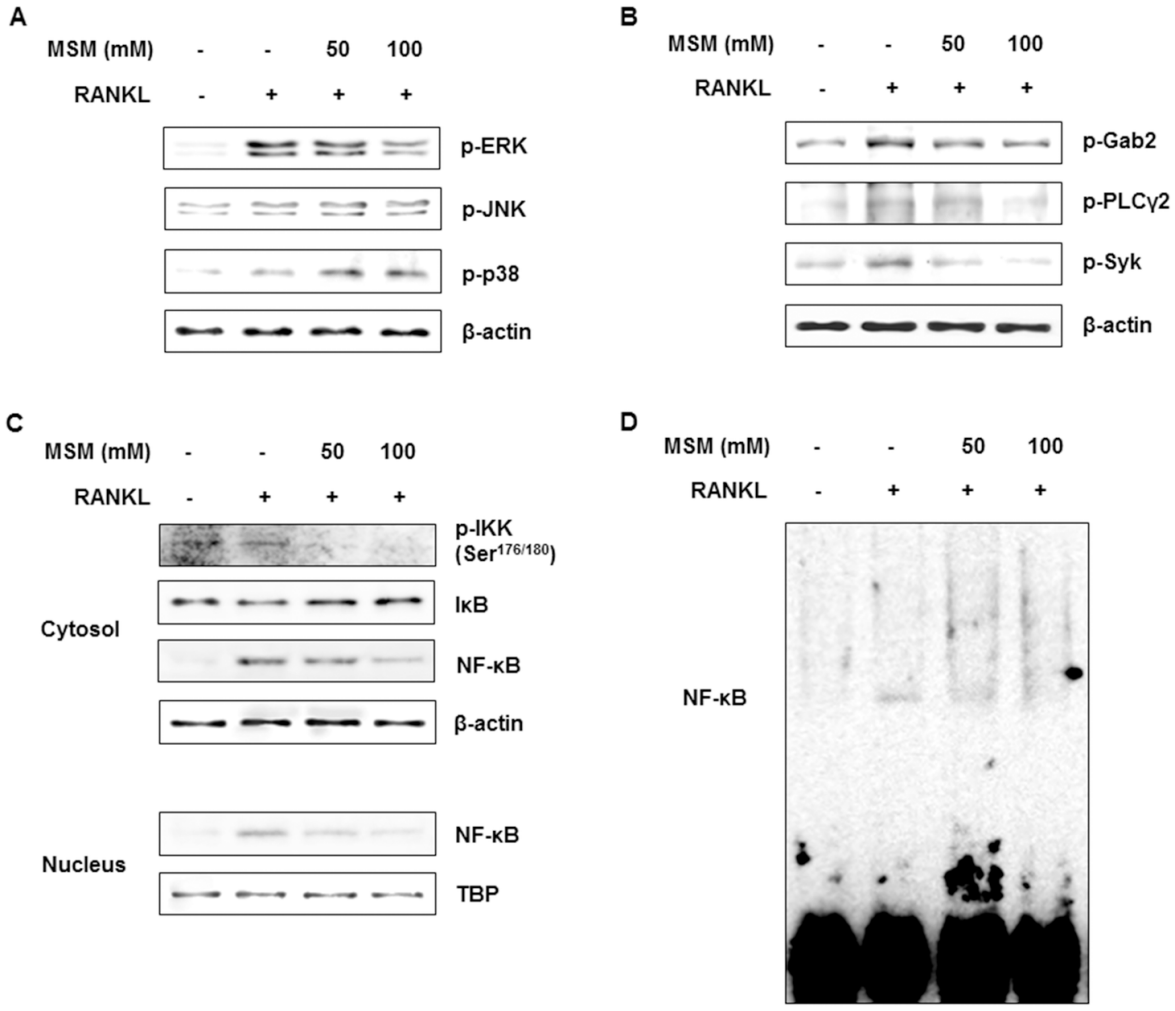
MSM inhibits RANKL-induced signaling in BMMs. BMMs were incubated with various concentration of MSM for 1 h, together with controls without MSM exposure, were then incubated with or without RANKL (100ng/ml) for 10 min. Cell lysates were immunoblotted for the indicated proteins. (A) MSM inhibits RANKL-induced activation of ERK. (B) MSM suppresses RANKL-induced activation of Gab2, PLCγ2, and Syk. (C) MSM suppresses RANKL-induced IKK phosphorylation, IκB degradation, and NF-κB activation. Tata binding protein (TBP) was used as nuclear protein loading control. (D) NF-κB DNA binding was detected by EMSA. Data shown are representative of three independent experiments.

We determined the effects of MSM on the activation of MAPK by RANKL in BMMs. MSM inhibited RANKL-induced phosphorylation of ERK in a dose-dependent manner but failed to diminish either JNK or p38 phosphorylation (Fig 3A). We then investigated the effect of MSM on the activation of PLCγ and Syk by RANKL in BMMs. RANKL-induced activation of both PLCγ and Syk were decreased by MSM in a dose-dependent manner (Fig 3B). These results suggested that MSM suppressed RANKL-induced osteoclastogenesis through blocking the expression of ITAM signaling molecules such as PLCγ and Syk.

To determine whether MSM suppresses the RANKL-induced activity of transcription factors by blocking NF-κB, we examined the effects of MSM on RANKL-induced NF-κB activation. As shown Fig 3C, MSM reduced RANKL-induced IKK phosphorylation and IκBα degradation in a dose-dependent manner. We also found that MSM significantly reduced RANKL-induced NF-κB signaling with diminished DNA binding of NF-κB as revealed by EMSA. These results demonstrated that MSM inhibited RANKL-stimulated osteoclastogenesis by blocking the activation of NF-κB, an essential factor for osteoclast differentiation.

### MSM Attenuates RANKL-Induced Osteoclastic Marker Gene Expression by Blocking STAT3 Activity

**Fig 4:**
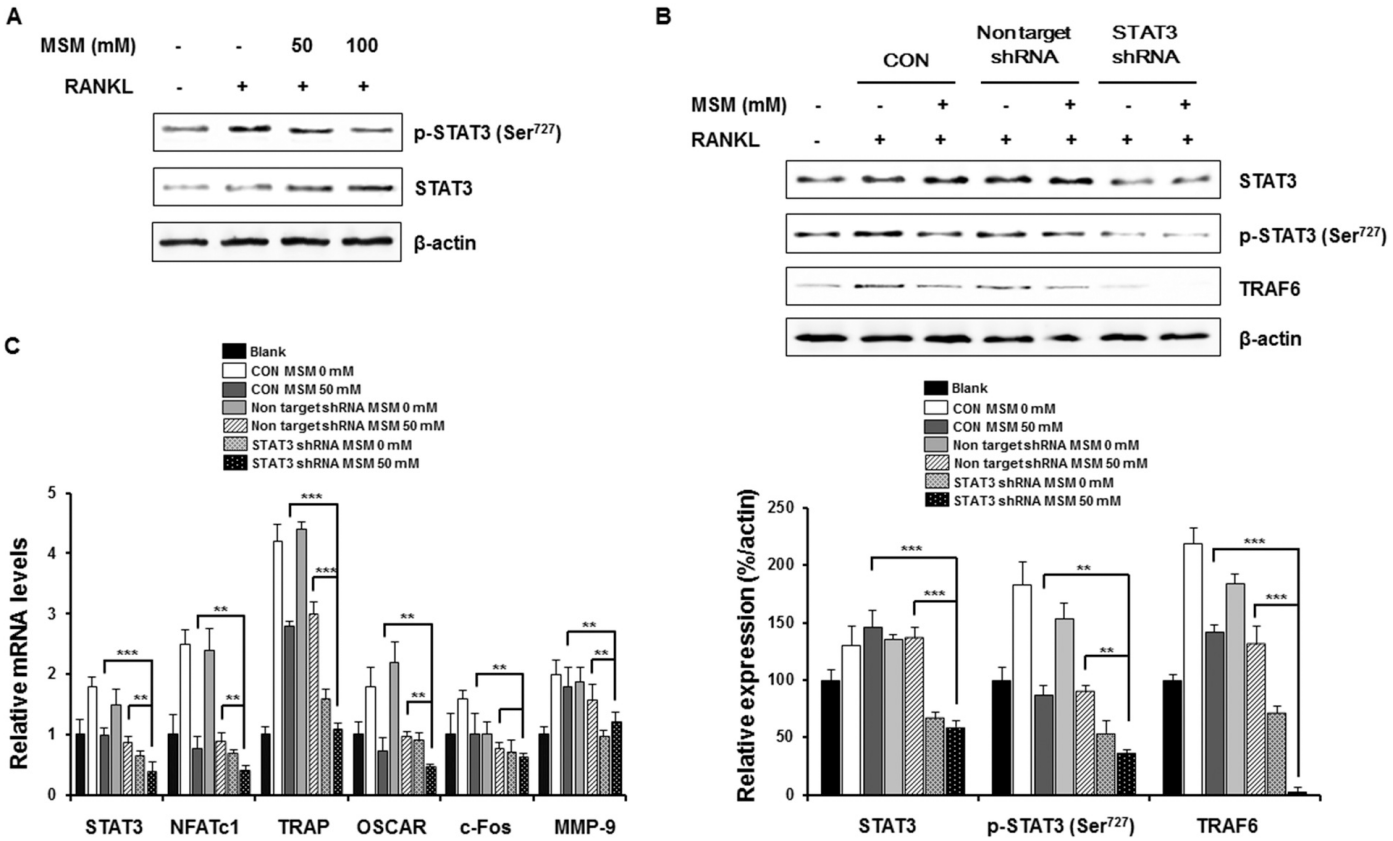
MSM attenuates RANKL-induced osteoclastic marker gene expression by blocking STAT3. (A) RAW264.7 cells were incubated with or without MSM for 1 h and then either exposed (or not) to RANKL (100ng/ml) for 10 min. Cell lysates were then blotted and immunostained with p-STAT3 and STAT3 antibodies. (B) RAW264.7 cells were transfected with STAT3 shRNA or a non-targeting shRNA for 48 h, then stimulated with RANKL (100 ng/ml) for 10 min. Cell lysates were prepared for western blot with antibodies as indicated. The relative levels of protein were determined using densitometry and normalized to β-actin. (C) RAW264.7 cells were transfected as in (B) and then stimulated with RANKL (100 ng/ml) for 24 h, with total RNA isolated using Qiagen. Expression of osteoclastic marker genes and STAT3 were examined using real-time PCR with GAPDH used as an internal control. Data shown are representative of three independent experiments. Asterisks indicate a significant increase by t-test (**p <0.01, ***p < 0.001).

Many studies have demonstrated the importance of STAT3 in bone physiology [12]. To investigate the effect of MSM on RANKL-induced phosphorylation of STAT3 we quantified the phosphorylation of Ser^727^ STAT3 by western blot. As expected, MSM inhibited RANKL-induced phosphorylation of Ser^727^ STAT3 (Fig 4A). To examine whether STAT3 is involved in RANKL-induced osteoclastogenesis we then used shRNA to target STAT3. As shown in Fig 4B, the shRNA construct significantly decreased the expression of STAT3, with diminished RANKL-induced phosphorylation of Ser^727^ STAT3, and TRAF6.

We then explored the role of STAT3 in RANKL-induced osteoclast marker gene expression using control and STAT3 specific shRNAs. As shown in Fig 4C, the mRNA levels of STAT3, together with various osteoclastogenic marker genes substantially decreased by the shRNA-mediated STAT3 knockdown. Collectively, these results demonstrate that STAT3 plays a pivotal role in RANKL-induced osteoclast formation and that MSM attenuated RANKL-induced osteoclastic marker gene expression by blocking STAT3 activity.

## Discussion

MSM is a low molecular weight organic sulfur compound with anti-oxidant and anti-inflammatory activities [13]. We recently found that MSM enhances osteoblast differentiation in MSCs through activation of STAT5b. Moreover, in osteoblast-like cells MSM induced GH signaling through the Jak2/STAT5b pathway [8]. However, the effects of MSM have yet to be reported for osteoclasts or their differentiation. Our results showed that MSM inhibits RANKL-induced osteoclastogenesis by suppressing NF-κB and STAT3 activities in BMMs.

In order to further investigate the inhibitory effect of MSM in BMM, we tested the influence of MSM on viability and osteoclast differentiation *in-vitro*. Our results showed that MSM inhibits RANKL-induced osteoclast differentiation without causing any significant decrease in viability of BMMs. Therefore, MSM exerted an inhibitory effect on RANKL-induced osteoclastogenesis.

In RANKL-induced signaling, the cytoplasmic domain of RANK recruits adaptor molecules such as the TRAF6 to initiate a signaling cascade [14]. TRAF6 is also involved in the activation of transcription factors such as NF-κB, NFATc1, and c-Fos [15]. Intriguingly, MSM significantly suppressed RANKL-induced expression of osteoclast marker genes, including TRAF6, c-Fos, NFATc1, and Cts K. MSM also inhibited the expression of OSCAR, which is induced by NFATc1.

The MAPKs (ERK, JNK, and p38) have been reported to be activated by RANKL stimulation and to be associated with osteoclastogenesis [4]. In this study, we evaluated the effects of MSM on the activation of MAPKs and identified a dose-dependent suppression of ERK but not p38 or JNK phosphorylation. ERK is known to induce c-Fos during osteoclastogenesis with its inhibition shown to decrease osteoclast formation [16]. These results tentatively suggest that MSM may contribute to the suppression of NF-κB and calcium signaling primarily, rather than MAPK activity. In addition to RANKL-induced activation of TRAF6, ITAM-activated co-stimulatory signals regulate osteoclastogenesis via cross-talk with RANK-induced signaling [17]. Phosphorylated ITAMs (induced by RANKL) serve as docking sites for the SH2 containing signaling molecule Syk, which then activates the PLCγ-calcium pathway, eventually leading to activation of NFATc1 [18]. As expected, MSM inhibited both Syk phosphorylation and PLCγ, which are critical for the activation of calcium signaling. Furthermore, MSM-induced suppression of osteoclastogenesis would also appear to occur, at least in part, through inhibition of the adaptor molecule Gab2, which is rapidly phosphorylated upon RANK stimulation and associates with the cytoplasmic tail of RANK to regulate osteoclastogenesis [19].

MAPKs and NF-κB are the two main effectors in activating the NFATc1 promoter to encourage NFATc1 expression [20]. The classic NF-κB signaling pathway involves the activation of the inhibitor of κB (IκB) kinase (IKK) complex that then phosphorylates IκBs, targeting them for ubiquitin-dependent degradation [20]. Our results showed that MSM suppressed the phosphorylation of IKK, leading to reduced cytoplasmic degradation of IκB and the prevention of NF-κB’s DNA-binding activity. Our results also indicated that inhibition of the NF-κB pathway suppressed RANKL-stimulated induction of NFATc1.

STAT3 is critical to the growth, survival, and differentiation of cells. It was reported that for the induction of RANKL and osteoclast formation, STAT3 activation in osteoblastic cells is required [21]. Although the role of STAT3 in osteoclast biology is somewhat controversial, the protein inhibitor of activated STAT3 (PIAS3) has been shown to negatively regulate RANKL-mediated osteoclastogenesis [22]. Furthermore during RANKL induced osteoclastogenesis, both NFATc1 expression and STAT3 activation were inhibited by AG490 (Jak2 inhibitor) [12]. These results support our hypothesis that MSM inhibits RANKL-induced phosphorylation of STAT3 Ser^727^, showing that STAT3 plays a pivotal role in RANKL-induced osteoclast formation.

We previously reported that MSM induced osteoblast differentiation via the Jak2/STAT5b pathways in MSCs [8]. On the other hand, MSM reduced the osteoclastic differentiation of BMMs, as shown from Figs 1 to 4. RANKL promotes bone resorption whereas OPG is a “decoy receptor” that binds and neutralizes RANKL, thus inhibiting bone resorption [4]; osteoblast/stromal cells express both of these genes [23]. We know that, the osteoblast precursor cells produce RANKL and the mature osteoblasts secrete OPG. Concurrent to this, in this study also MSM induced the secretion of OPG and inhibited RANKL production in osteoblasts (Fig 2C). This suggests that MSM regulate osteoclastogenesis indirectly via MSCs, besides its direct regulation through inhibiting STAT3/TRAF6 signaling axis, and the differentiation and function of osteoclasts.

We demonstrated that MSM suppressed RANKL-induced osteoclastogenesis in BMMs by inhibiting the activation of NF-κB. MSM reduces RANKL-induced osteoclastic marker gene expression by blocking STAT3 activity. We verified that RANKL-induced osteoclastogenesis is dependent on the coordinated mechanisms of NF-κB and STAT3, as mediated by MSM. These data elaborate MSM’s mechanism of action in altering osteoclastogenesis and identify MSM as a potential therapeutic candidate for the treatment of disorders associated with bone loss.

## References

[1] Park H, Noh AL, Kang JH, Sim JS, Lee DS, Yim M. Peroxiredoxin II negatively regulates lipopolysaccharide-induced osteoclast formation and bone loss via JNK and STAT3. Antioxid Redox Signal. 2015;22:63–77. 10.1089/ars.2013.5748 25074339 PMC4270137

[2] Tanake Y, Nakayamada S, Okada Y. Osteoblasts and osteoclasts in bone remodeling and inflammation. Curr Drug Targets Inflamm Allergy. 2005;4:325–328. 16101541 10.2174/1568010054022015

[3] Park JS, Kwok SK, Lim MA, Kim EK, Ryu JG, Kim SM, et al STA-21, a promising STAT3 inhibitor that reciprocally regulates Th17 and treg cells, inhibits osteoclastogenesis in mice and humans and alleviates autoimmuno inflammation in an experimental model of rheumatoid arthritis. ARTHRITIS & RHEUMATOLOGY. 2014;66:918–929. 24757144 10.1002/art.38305

[4] Boyle WJ, Simonet WS, Lacey DL. Osteoclast differentiation and activation. Nature. 2003;423:337–342. 12748652 10.1038/nature01658

[5] Quan GH, Wang H, Cao J, Zhang Y, Wu D, Peng Q, et al Calycosin Suppresses RANKL-Mediated Osteoclastogenesis through Inhibition of MAPKs and NF-κB. Int J Mol Sci. 2015;16(12):29496–507. 10.3390/ijms161226179 26690415 PMC4691122

[6] Logar DB, Komadina R, Prezelj J, Ostanek B, Trost Z, Marc J. Expression of bone resorption genes in osteoarthritis and in osteoporosis. J Bone Miner Metab. 2007;25: 219–225. 17593491 10.1007/s00774-007-0753-0

[7] Jove R. Preface: STAT signaling. Oncogene. 2000;19:2466–7. 10851044 10.1038/sj.onc.1203549

[8] Joung YH, Lim EJ, Darvin P, Chung SC, Jang JW, Do Park K, et al MSM enhances GH signaling via the Jak2/STAT5b pathway in osteoblast-like cells and osteoblast differentiation through the activation of STAT5b in MSCs. PLoS One. 2012;7: e47477 10.1371/journal.pone.0047477 23071812 PMC3469535

[9] Kim K, Lee J, Kim JH, Jin HM, Zhou B, Lee SY, et al Protein inhibitor of activate STAT3 modulates osteoclastogenesis by down-regulation of NFATc1 and osteoclast-associated receptor. J Immunol. 2007;178:5588–5594. 17442941 10.4049/jimmunol.178.9.5588

[10] Duplomb L, Baud'huin M, Charrier C, Berreur M, Trichet V, Blanchard F, Heymann D. Interleukin-6 inhibits receptor activator of nuclear factor κB ligand-induced osteoclastoge nesis by diverting cells into the macrophage lineage: key role of serine^727^ phosphorylation of signal transducer and activator of transcription 3. Endocrinology. 2008;149:3688–3697. 10.1210/en.2007-1719 18403479

[11] Kim YH, Kim DH, Lim H, Baek DY, Shin HK, Kim JK. The anti-inflammatory effects of methylsulfonylmethane on lipopolysaccharide-induced inflammatory responses in murine macrophages. Biol Pharm Bull. 2009;32:651–656. 19336900 10.1248/bpb.32.651

[12] Li CH, Zhao JX, Sun L, Yao ZQ, Deng XL, Liu R, et al AG490 inhibits NFATc1 expression and STAT3 activation during RANKL induced osteoclastogenesis. Biochem Biophys Res Commun. 2013;435:533–539. 10.1016/j.bbrc.2013.04.084 23665018

[13] Horváth K, Noker PE, Somfai-Relle S, Glávits R, Financsek I, Schauss AG. Toxicity of methylsulfonylmethane in rats. Food Chem Toxicol. 2002;40:1459–1462. 12387309 10.1016/s0278-6915(02)00086-8

[14] Lamothe B, Webster WK, Gopinathan A, Besse A, Campos AD, Darnay BG. TRAF6 ubiquitin ligase is essential for RANKL signaling and osteoclast differentiation. Biochem Biophys Res Commun. 2007;359:1044–9. 17572386 10.1016/j.bbrc.2007.06.017 PMC2732028

[15] Feng X. Regulatory roles and molecular signaling of TNF family members in osteoclasts. Gene. 2005;350:1–13. 15777737 10.1016/j.gene.2005.01.014

[16] Monje P, Hernández-Losa J, Lyons RJ, Castellone MD, Gutkind JS. Regulation of the transcriptional activity of c-Fos by ERK. A novel role for the prolyl isomerase PIN1. See comment in PubMed Commons below J Biol Chem. 2005;280:35081–4. 10.1074/jbc.C500353200 16123044

[17] Xu Feng. RANKing intracellular signaling in osteoclasts. IUBMB Life. 2005;57:389–395. 16012047 10.1080/15216540500137669

[18] Koga T, Inui M, Inoue K, Kim S, Suematsu A, Kobayashi E, et al Costimulatory signals mediated by the ITAM motif cooperate with RANKL for bone homeostasis. Nature. 2004;428:758–763. 15085135 10.1038/nature02444

[19] David JP, Sabapathy K, Hoffmann O, Idarraga MH, Wagner EF. JNK1 modulates osteoclasttogenesis through both c-Jun phosphorylation-dependent and -independent mechanisms. J Cell Sci. 2002:115;4317–4325. 12376563 10.1242/jcs.00082

[20] Asagiri M, Takayanagi H. The molecular understanding of osteoclast differentiation. Bone. 2007;40:251–264. 17098490 10.1016/j.bone.2006.09.023

[21] O'Brien CA, Gubrij I, Lin SC, Saylors RL, Manolagas SC. STAT3 activation in stromal/osteoblastic cells is required for induction of the receptor activator of NF-kappaB ligand and stimulation of osteoclastogenesis by gp130-utilizing cytokines or interleukin-1 but not 1,25-dihydroxyvitamin D3 or parathyroid hormone. J Biol Chem. 1999;274:19301–8. 10383440 10.1074/jbc.274.27.19301

[22] Hikata T, Takaishi H, Takito J, Hakozaki A, Furukawa M, Uchikawa S, et al PIAS3 negatively regulates RANKL-mediated osteoclastogenesis directly in osteoclast precursors and indirectly via osteoblasts. Blood. 2009;113:2202–12. 10.1182/blood-2008-06-162594 18952894 PMC3401028

[23] Khosla S. The OPG/RANKL/RANK system. Endocrinology. 2001;142:5050–5055. 11713196 10.1210/endo.142.12.8536

